# Insights about serum sodium behavior after 24 hours of continuous renal replacement therapy

**DOI:** 10.5935/0103-507X.20160026

**Published:** 2016

**Authors:** Thiago Gomes Romano, Cassia Pimenta Barufi Martins, Pedro Vitale Mendes, Bruno Adler Maccagnan Pinheiro Besen, Fernando Godinho Zampieri, Marcelo Park

**Affiliations:** 1 Intensive Care Unit, Hospital Sírio-Libanês - São Paulo (SP), Brazil.; 2 Discipline of Nephrology, Faculdade de Medicina do ABC - Santo André (SP), Brazil.; 3 Intensive Care Unit, Hospital Baia Sul - Florianópolis (SC), Brazil.; 4 Intensive Care Unit, Department of Emergency, Hospital das Clínicas, Faculdade de Medicina, Universidade de São Paulo - São Paulo (SP), Brazil.

**Keywords:** Renal replacement therapy, Hemofiltration, Hemodiafiltration, Sodium, Critical care

## Abstract

**Objective:**

The aim of this study was to investigate the clinical and laboratorial factors associated with serum sodium variation during continuous renal replacement therapy and to assess whether the perfect admixture formula could predict 24-hour sodium variation.

**Methods:**

Thirty-six continuous renal replacement therapy sessions of 33 patients, in which the affluent prescription was unchanged during the first 24 hours, were retrieved from a prospective collected database and then analyzed. A mixed linear model was performed to investigate the factors associated with large serum sodium variations (≥ 8mEq/L), and a Bland-Altman plot was generated to assess the agreement between the predicted and observed variations.

**Results:**

In continuous renal replacement therapy 24-hour sessions, SAPS 3 (p = 0.022) and baseline hypernatremia (p = 0.023) were statistically significant predictors of serum sodium variations ≥ 8mEq/L in univariate analysis, but only hypernatremia demonstrated an independent association (β = 0.429, p < 0.001). The perfect admixture formula for sodium prediction at 24 hours demonstrated poor agreement with the observed values.

**Conclusions:**

Hypernatremia at the time of continuous renal replacement therapy initiation is an important factor associated with clinically significant serum sodium variation. The use of 4% citrate or acid citrate dextrose - formula A 2.2% as anticoagulants was not associated with higher serum sodium variations. A mathematical prediction for the serum sodium concentration after 24 hours was not feasible.

## Abstract (pt)

**Objetivo:**

Investigar os fatores clínicos e laboratoriais associados com a variação dos níveis séricos de sódio durante terapia renal substitutiva contínua e avaliar se a fórmula de mixagem perfeita pode prever a variação do sódio nas 24 horas.

**Métodos:**

A partir de uma base de dados coletada de forma prospectiva, recuperamos e analisamos os dados referentes a 36 sessões de terapia renal substitutiva realizadas em 33 pacientes, nas quais a prescrição de afluentes permaneceu inalterada durante as primeiras 24 horas. Aplicamos um modelo linear misto para investigar os fatores associados com grandes variações dos níveis séricos de sódio (≥ 8mEq/L) e geramos um gráfico de Bland-Altman para avaliar a concordância entre as variações previstas e observadas.

**Resultados:**

Nas sessões de terapia renal substitutiva de 24 horas identificamos que SAPS 3 (p = 0,022) e hipernatremia basal (p = 0,023) foram preditores estatisticamente significantes de variações séricas do sódio ≥ 8mEq/L na análise univariada, porém apenas hipernatremia demonstrou uma associação independente (β = 0,429; p < 0,001). A fórmula de mixagem perfeita para previsão do nível de sódio após 24 horas demonstrou baixa concordância com os valores observados.

**Conclusões:**

A presença de hipernatremia por ocasião do início da terapia renal substitutiva é um fator importante associado com variações clinicamente significativas dos níveis séricos de sódio. O uso de citrato 4% ou da fórmula A de ácido citrato dextrose 2,2% como anticoagulantes não se associou com variações mais acentuadas dos níveis séricos de sódio. Não foi viável desenvolver uma predição matemática da concentração do sódio após 24 horas.

## INTRODUCTION

Continuous renal replacement therapy (CRRT) is a widely adopted supportive therapy that is used in critically ill patients. Although definitive indications for CRRT are controversial, its use should be strongly considered in specific situations, such as severe hemodynamic instability and brain edema.^(1-3)^ In these situations, blood osmolality variation potentially causes serious adverse effects, which include disequilibrium syndrome, osmotic demyelination, brain edema and hypotension.^(4,5)^

The serum sodium concentration is a major determinant of blood osmolality, and its variation over time is extremely important.^(6)^ A large 24-hour variation of the serum sodium concentration in the presence of either hyponatremia or hypernatremia is associated with encephalic derangements (brain edema or osmotic demyelination syndrome), and to avoid such derangements, a variation smaller than 8mEq/L is considered safe.^(5,7)^ In addition, citrate anticoagulation is the preferred method for filter protection during CRRT compared to heparin,^(8)^ but its use may be associated with sodium variations that are often unpredictable. Currently, scheduled laboratory monitoring and fine tuning have been standard procedures to modulate serum electrolytes until achieving the stable target value.^(9)^ Furthermore, there are no validated prediction rules to estimate sodium variation during CRRT with citrate as the filter protection method.

Thus, the primary objectives of this study were to investigate whether citrate anticoagulation is associated with large serum sodium variations over 24 hours and whether the perfect admixture formula could predict its variation. Secondary objectives were to assess the clinical and laboratorial factors associated with a large serum sodium variation and to additionally explore predictors of the resulting serum sodium concentration up to 24 hours of continuous renal replacement therapy.

## METHODS

Continuous renal replacement therapy sessions were retrieved from two prospectively collected electronic databases from two hospitals (*Hospital Sírio Libanês* and *Hospital das Clínicas da Faculdade de Medicina de São Paulo*) located in São Paulo, Brazil. These data were reviewed from 2003 to 2012 in one hospital and from 2011 to 2012 in another hospital. A CRRT session was retrieved if the session had lasted for at least 24 hours and if the affluent fluid prescription was not modified during this time period. Two CRRT machines were used during this study, a Diapact^®^ CRRT (BBraun Laboratories, Melsungen, Germany) and Prismaflex^®^ System (Gambro, Lund, Sweden).

This study protocol followed the guidelines of the Declaration of Helsinki. The institutional review board of the hospital (*Comissão para Análise de Projetos de Pesquisa* - CAPPesq) reviewed and approved this study (CAPPesq protocol number 107443). The requirement for written informed consent was waived because there was no intervention; we used a database that assured patient confidentiality.

Clinical and laboratorial data were collected from the electronic records. When necessary, laboratorial data were retrieved from the electronic consulting system. Data are shown for the whole group (all CRRT sessions) and were categorized into two groups: The first group (Group 1) underwent CRRT sessions with 24 hours of serum sodium concentration variation ≥ |8|mEq/L; and the second group (Group 2) with 24 hours of serum sodium concentration variation < |8|mEq/L.

### Continuous renal replacement therapy initiation and conduction

In both units, the CRRT prescription was individualized according to the patients' clinical situation. To enhance filter protection, 4% citrate was routinely used in one hospital, 2.2% acid citrate dextrose - formula A (ACD-A) was used in the other hospital, and both hospitals commonly used heparin and a normal saline lavage when necessary. Post-filtration and systemic ionized calcium and sodium activated the partial thromboplastin time (aPTT), and potassium, venous blood pH, bicarbonate and standard base excess (SBE) were routinely collected, as necessary, every six hours. The initial sodium affluent concentration prescription was based on the serum concentration, presence of brain edema and anticoagulant use. With the use of heparin or lavage, the sodium concentration was prescribed to equalize the targeted serum concentration. The electrolyte composition of the fluid replacement was identical to the dialysate in cases of continuous venous-venous hemodiafiltration (CVVHDF). When using 4% citrate and 2.2% ACD-A, sodium and bicarbonate concentrations were prescribed at 15 - 20mEq/L and 5 - 10mEq/L below the targeted serum concentration, respectively, because the 4% citrate solution contained 408mEq/L of sodium and the 2.2% ACD-A solution contained 224mEq/L of sodium.

Next, 4% citrate was initially prescribed at a flow rate of 40 - 50 units below the blood flow (using different units, mL/hour to the 4% citrate flow and mL/minute to the blood flow). Furthermore, 2.2% ACD-A was initially prescribed at a rate of 1.5 times the blood flow. Elementary calcium was replaced at a rate of 1 - 2mg/kg/hour using chloride or gluconate. The anticoagulants and elementary calcium infusion were then adjusted according to the collected aPTT or ionized calcium (systemic and/or post-filter) according to standardized protocols.

### Serum sodium prediction after 24 hours

The perfect admixture formula, which was originally presented in this manuscript, takes into account that all of the components that pass through the filter have robust diffusibility and sieving properties, including 4% citrate^(10)^ and 2.2% ACD-A.^(11)^ Consequently, the serum sodium in the CRRT venous line consisted of the respective flow proportional admixture of the anticoagulant, affluent fluid and blood, which occurs in the filter and in the venous line ("mixing chamber"). This finding justifies the reduction in the affluent fluid sodium concentration during CRRT using 4% citrate^(12)^ or 2.2% ACD-A^(13)^ as an anticoagulant to achieve systemic equilibrium without hypernatremia. The 4% citrate and 2.2% ACD-A had a sodium concentration of 408mEq/L and 224mEq/L, respectively. Furthermore, the resultant venous line sodium concentration was tightly associated with the equilibrated systemic serum sodium concentration during CRRT.^(14)^ Thus, we assumed that serum sodium after 24 hours resulted from the equilibrium of the proportional admixture of sodium derived from the affluent, blood and anticoagulant.

**Figure 1:**
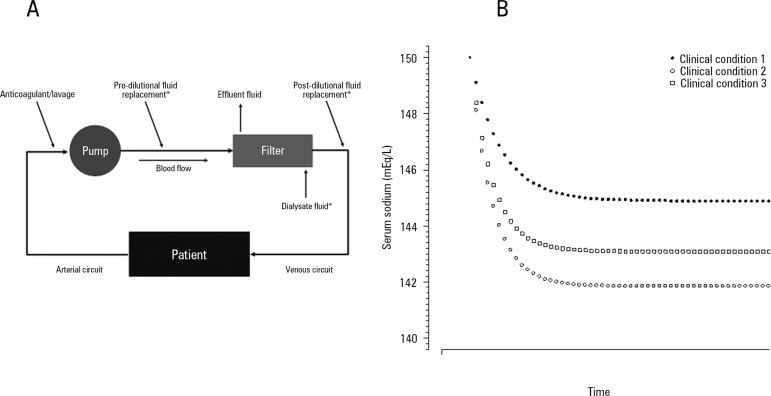
Serum sodium prediction using the total admixture formula. A) Principles of the formula, where the filter and continuous renal replacement therapy venous circuit are assumed to be a “perfect” mixing chamber, and 4% citrate and 2.2% ACD-A are considered with a sieving coefficient ~1 and diffusibility ~1 through the filter. B) Equilibrium of the serum sodium through the time of continuous renal replacement therapy in three different clinical conditions. All three conditions started with a serum sodium = 150mEq/L, affluent sodium = 135mEq/L, and a blood flow = 180mL/minute. The three different clinical conditions were: clinical condition 1: continuous renal replacement therapy dosage = 2000mL/hour, and 2.2% ACD-A (224mEq/L of sodium) flow = 250mL/hour; Clinical condition 2: continuous renal replacement therapy dosage = 3000mL/hour, and 2.2% ACD-A flow = 250mL/hour; Clinical condition 3: continuous renal replacement therapy dosage = 2000mL/hour, and 2.2% ACD-A flow = 300mL/hour. * Only used in continuous venous-venous hemofiltration and continuous venous-venous hemodiafiltration; # Only used in continuous venous-venous hemodialysis and continuous venous-venous hemodiafiltration.

Figure 1A shows the principle of the perfect sodium admixture among the cited components, in which the filter and venous circuit are a mixing chamber. Using this mixing chamber principle, we hypothesized that administration of the perfect admixture back into the patient will determine the final serum sodium concentration. The equilibrium process requires an unpredictable amount time, as shown in figure 1 B.

For each milliliter of blood passing through the filter, the perfect admixture principle can be mathematically written as follows (sodium units are mEq):

### Sodium mass

Blood sodium (Bs) = Systemic serum sodium concentration * blood flow/1000

Affluent fluid sodium (As) = Affluent sodium concentration * dosage (in L/hour)/1000 * 60

Anticoagulant sodium (ACs) = Sodium concentration (408mEq/L of sodium for 4% citrate and 224mEq/L for the 2.2% ACD-A solution) * ACs flow (in mL/hour)/60

### Diluents' volume

Blood volume (Bv) = Blood flow (mL/minute)

Affluent volume (Av) = Dialysis dose (mL/hour)/60

Anticoagulant volume (ACv) = Anticoagulant flow (mL/hour)/60

### The sodium inside the mixing chamber is:

[Na^+^] = (Bs + As + ACs)/(Bv + Av + Acv)

The Bs value is renewed after each cycle of serum sodium equilibrium, resulting in a non-linear variation of serum sodium until equilibrium, as shown in figure 1 A.

However, this sodium concentration will equilibrate in serum and re-enter the CRRT machine, leading to a new Bs value, and thus, a new value of equilibrium will be achieved until the final value is obtained.

### Statistical analysis

Data were predominantly non-parametrically distributed (as tested using the Shapiro-Wilk goodness-of-fit model), and thus, they are presented as the median [25% - 75%], except for the difference between observed and predicted sodium, which was shown as the mean and standard deviation. Comparisons between different groups were performed using the Mann-Whitney test, and comparisons of subjects of the same group over time were performed using the Friedman test. *Post-hoc* analyses were not performed because temporal tests are only used to demonstrate trends. A mixed linear model using the CRRT session as the random factor was used to investigate the independent association between SAPS 3 and initial serum sodium with the 24-hour serum sodium variation as the dependent variable because the aforementioned two variables were significantly different between groups at baseline. A Bland-Altman diagram was generated to demonstrate agreements between predicted sodium concentration at 24 hours using the perfect admixture formula and observed values at 24 hours.^(15)^ The R-free source software was used to performed all of the statistical analyses and to generate graphs.^(16)^

## RESULTS

A total of 112 sessions of CRRT were reviewed, and 36 CRRT sessions on 33 patients were retrieved. Only continuous venous-venous hemofiltration (CVVH) and CVVHDF were prescribed. The general clinical data of the whole group are shown in table 1. Group 1 received 7 sessions (7 patients) and Group 2 received 29 sessions (26 patients). Table 2 shows the clinically and metabolically relevant data immediately before CRRT initiation, and the amount of serum sodium concentration variation during the 24-hour session was also analyzed. These two tables showed that the disease severity at ICU admission (disclosed by the SAPS 3 score) and initial serum sodium concentration were slightly different between Groups 1 and 2. The more severe the hypernatremia, the more intense the serum sodium variation. Multivariate analysis revealed that only the initial sodium was significantly related (beta coefficient = 0.429, p < 0.001) to the sodium variation during the first 24 hours of CRRT, while disease severity (SAPS 3) was not related (beta coefficient = - 0.050, p = 0.615).

**Table 1:** Characteristics of the whole group of 33 patients analyzed and the groups categorized according to the 24 hours sodium variation ≥ |8|mEq/L or <|8|mEq/L

	**Whole group** ** (N = 33 patients)**	**[Na^+^] 24 hours variation ≥ |8|mEq/L (N = 7 patients)**	**[Na^+^] 24 hours variation < |8|mEq/L** ** (N = 26 patients)**	p value*
Patients characteristics				
Age (years)	63 (52 - 80)	58 (39 - 80)	64 (57 - 80)	0.597
SAPS 3	55 (54 - 56)	54 (42 - 55)	56 (55 - 57)	0.022
Female gender	4 (12)	1 (14)	3 (12)	0,754
Admission SOFA	8.5 [8.5 - 8.5]	8.5 [8.5 - 10.3]	8.5 [8.5 - 8.5]	0.474
Weight (kg)	68 [60 - 78]	73 [67 - 77]	68 [60 - 80]	0.343
Height (cm)	165 [164 - 171]	170 [169 - 180]	165 [163 - 170]	0.095
Pre-ICU admission LOS (days)	2.0 [1.0 - 7.0]	1.0 [0.5 - 2.5]	2.0 [1.0 - 7.8]	0.503
Diagnosis				
Septic shock	25 (76)	4 (57)	21 (81)	0.320
Cardiogenic shock	4 (12)	2 (29)	2 (8)	0.190
Hypovolemic shock	1 (3)	0 (0)	1 (4)	1.000
Multiple trauma	1 (3)	1 (14)	0 (0)	0.212
Respiratory failure	1 (3)	0 (0)	1 (4)	1.000
Metabolic encephalopathy	1 (3)	0 (0)	1 (4)	1.000
Comorbidities				
Chronic hypertension	21 (64)	2 (28)	19 (73)	0.071
Chronic renal failure	14 (42)	2 (28)	12 (46)	0.670
Diabetes	11 (33)	1 (14)	10 (38)	0.378
Coronary heart disease	11 (33)	2 (28)	9 (35)	1.000
Heart failure	7 (21)	1 (14)	6 (23)	1.000
COPD	3 (9)	0 (0)	3 (12)	1.000
Cirrhosis	1 (3)	1 (14)	0 (0)	0.212
ICU support#				
Inotropes	16 (48)	1 (14)	15 (58)	0.085
Vasopressors	26 (79)	5 (71)	21 (81)	0.623
Mechanical ventilation	23 (70)	6 (86)	17 (65)	0.397
Antibiotics	33 (100)	7 (100)	26 (100)	1.000
Outcomes				
ICU survival	21 (64)	4 (57)	17 (65)	0.686
Hospital survival	21 (64)	4 (57)	17 (65)	0.686

Na^+^ - sodium; SAPS - Simplified Acute Physiological Score; SOFA - Sequential Organ Failure Assessment; ICU - intensive care unit; LOS - length of stay; COPD - chronic obstructive pulmonary disease.

* p value of the comparison between the groups with sodium variation ≥ |8|mEq/L and < |8|mEq/L within the first 24 hours.

# At any time of the intensive care unit stay. The results are expressed as the median (25% - 75%) or as the number (%).

**Table 2:** Clinical, laboratorial and continuous renal replacement therapy data immediately before initiation

	**Whole group** ** (N = 36 sessions)**	**[Na^+^] 24 hours variation** **≥ |8|mEq/L** ** (N = 7 sessions)**	**[Na^+^] 24 hours variation** ** < |8|mEq/L** ** (N = 29 sessions)**	p value*
Laboratorial data				
pH	7.36 [7.29 - 7.40]	7.35 [7.29 - 7.38]	7.37 [7.29 - 7.40]	0.537
PaCO_2_ (mmHg)	40 [33 - 48]	36 [33 - 45]	41 [34 - 48]	0.508
HCO_3_^-^ (mEq/L)	21 [15 - 24]	21 [19 - 22]	21 [14 - 25]	0.912
SBE (mEq/L)	-3.3 [-8.9 - -0.4]	-4.5 [-6.4 - -2.4]	-3.0 [-9.3 - -0.4]	0.842
Lactate^-^ (mEq/L)	2.4 [1.6 - 3.2]	2.4 [2.3 - 2.9]	2.5 [1.4 - 3.3]	0.646
Na^+^ (mEq/L)	140 [136 - 145]	151 [141 - 161]	140[136 - 144]	0.023
K^+^ (mEq/L)	4.5 [4.1 - 4.9]	4.4 [3.9 - 4.9]	4.5 [4.1 - 4.9]	0.895
Cl^-^ (mEq/L)	105 [101, 109]	110 [106 - 131]	103 [100 - 108]	0.074
Na^+^ - Cl^-^	36.0 [32.0 - 40.0]	34.0 [32.5 - 39.0]	36.0 [32.2 - 40.0]	0.674
Na^+^ variation - during 24 hours (mEq/L)	1.0 [-2.0 - 5.0]	15.0 [10.5 - 17.0]	1.5 [0.0 - 6.5]	< 0.001
Creatinine (mg/dL)	2.62 [1.73 - 4.93]	2.62 [1.58 - 5.32]	2.62 [1.77 - 3.72]	0.929
Dysnatremia severity class				
Na^+^ < 125mEq/L	0 (0)	0 (0)	0 (0)	-----
Na^+^ < 135mEq/L	6 (17)	0 (0)	6 (21)	0.301
Na^+^ ≥ 135mEq/L and ≤ 145mEq/L	19 (53)	3 (43)	16 (55)	0.422
Na^+^ > 145mEq/L	8 (22)	4 (57)	4 (14)	0.030
Na^+^ > 160mEq/L	2 (6)	2 (29)	0 (0)	0.033
CRRT prescription				
CVVH	30 (84)	7 (100)	23 (79)	1.000
Post-dilutional replacement	0 (0)	0 (0)	0 (0)	-----
Pre-dilutional replacement	28 (93)	7 (100)	21 (91)	1.000
Hybrid replacement†	2 (7)	0 (0)	2 (9)	1.000
CVVHD	0 (0)	0 (0)	0 (0)	-----
CVVHDF	6 (16)	0 (0)	6 (21)	0.317
Post-dilutional replacement	0 (0)	0 (0)	0 (0)	-----
Pre-dilutional replacement	6 (100)	0 (0)	6 (100)	1.000
Hybrid replacement†	0 (0)	0 (0)	0 (0)	-----
Citrate 4% use	20 (56)	3 (43)	17 (59)	0.675
ACD-A 2.2% use	8 (22)	3 (43)	5 (17)	0.167
Heparin use	2 (6)	1 (14)	1 (3)	0.356
Lavage use	6 (16)	0 (0)	6 (79)	0.317
Dosage (mL/kg/hour)‡	35 [28 - 44]	35 [27 - 43]	35 [28 - 44]	0.952
Clinical data				
Cumulative fluid balance (mL)	3800 [1000 - 5400]	3800 [1050 - 5949]	3800 [1025 - 5300]	0.877
Diuresis of the day before initiation (mL)	500 [340 -1130]	690 [415 - 1420]	500 [310 - 830]	0.495

Na^+^ - sodium; PaCO_2_ - partial pressure of carbon dioxide; HCO_3_ - bicarbonate; SBE - standard base excess; K^+^ - potassium; Cl^-^ - chloride; Na^+^ - Cl^-^ - sodium-chloride; CRRT - continuous renal replacement therapy; CVVH - continuous venous-venous hemofiltration; CVVHD - continuous venous-venous hemodialysis; CVVHDF - continuous venous-venous hemodiafiltration; ACD-A - acid citrate dextrose - formula A.

† In these sessions, the pre- and post-dilutional fluid replacement were used at the same time during continuous venous-venous hemofiltration and continuous venous-venous hemodiafiltration (Both sessions used the ratio pre/post dilutional = 2/1).

* p value of the comparison between the groups with sodium variation ≥|8|mEq/L and <|8|mEq/L within the first 24 hours.

‡ Calculated using the effluent flow rate. The results are expressed as the median (25% - 75%) or as number (%).

**Figure 2:**
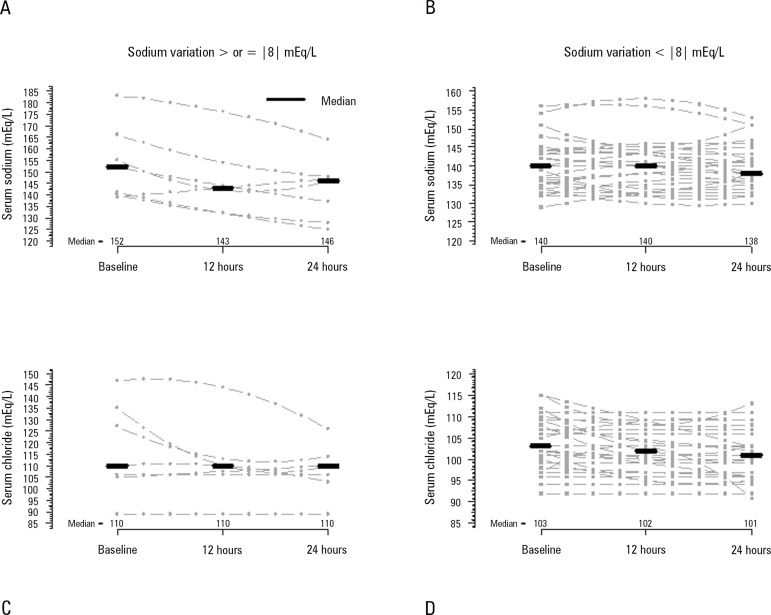
Sodium and chloride serum concentrations over the first 24 hours after continuous renal replacement therapy initiation. A and B) Sodium concentrations in the groups with sodium variations ≥ |8|mEq/L and < |8|mEq/L, respectively. The sodium concentration of Friedman´s analysis resulted in p = 0.040 in panel A and p = 0.841 in panel B. C and D) Chloride concentration in the groups with sodium variation ≥ |8|mEq/L and < |8|mEq/L, respectively. The chloride concentration of Friedman´s analysis resulted in p = 0.486 in panel C and p < 0.001 in panel D.

Figure 2 shows the temporal behavior of serum sodium and chloride during the first 24 hours, and tables 3 and 4 show the same temporal range behavior of the other patients' relevant metabolic variables and CRRT data, respectively. Importantly, no patient received hypertonic solutions during the CRRT session. The serum sodium median variations between 24 hours after CRRT initiation and baseline were 0.0 [0.3 - 2.0], -1.0 [-5.0 - 2.0], and -3.0 [-7.5 - -1.0] mEq/L (p = 0.280) in patients who were anticoagulated with heparin, 4% citrate and 2.2% ACD-A, respectively. Metabolic acidosis improved during the CRRT session, and PaCO_2_ also increased. CRRT related variables were relatively stable.

**Table 3:** Metabolic data variation within the first 24 hours of continuous renal replacement therapy

	**[Na^+^] 24 hours variation**	**CRRT initiation**	**12 hours**	**24 hours**	p value*
pH	Whole group	7.37 [7.29 - 7.40]	7.34 [7.27 - 7.42]	7.40 [7.34 - 7.43]	0.072
	≥ |8|mEq/L	7.34 [7.32 - 7.35]	7.37 [7.31 - 7.43]	7.37 [7.31 - 7.40]	0.717
	< |8|mEq/L	7.37 [7.28 - 7.42]	7.34 [7.27 - 7.42]	7.40 [7.34 - 7.44]	0.132
PaCO_2_ (mmHg)	Whole group	37 [30 - 47]	41 [33 - 45]	41 [37 - 46]	0.014
	≥ |8|mEq/L	35 [28 - 42]	37 [32 - 43]	45 [38 - 48]	0.018
	< |8|mEq/L	39 [31 - 47]	41 [33 - 46]	40 [37 - 45]	0.580
SBE (mEq/L)	Whole group	-3.7 [-9.0 - -1.0]	-3.4 [-7.7 - 1.1]	-0.8 [-4.4 - 1.7]	< 0.001
	≥ |8|mEq/L	-6.3 [-9.1 - -2.5]	-1.3 [-7.6 - 1.1]	0.6 [-1.4 - 1.2]	0.236
	< |8|mEq/L	-3.4 [-8.7 - -0.7]	-4.0 [-7.2 - 0.6]	-1.2 [-4.2 - 2.0]	0.095
HCO_3_^-^ (mEq/L)	Whole group	21 [15 - 24]	22 [18 - 25]	23 [20 - 26]	< 0.001
	≥ |8|mEq/L	19 [14 - 23]	22 [18 - 25]	25 [22 - 27]	0.169
	< |8|mEq/L	21 [15 - 24]	22 [18 -25]	23 [20 - 26]	0.099
Lactate^-^ (mEq/L)	Whole group	2.3 [1.4 - 3.2]	2.3 [1.7 - 3.5]	2.2 [1.7 - 3.1]	0.088
	≥ |8|mEq/L	2.9 [2.2 - 3.7]	1.3 [0.9 - 1.8]	2.0 [1.8 - 2.2]	0.097
	< |8|mEq/L	2.3 [1.4 - 3.2]	2.7 [1.9 - 3.7]	2.3 [1.7 - 3.2]	0.033
K^+^ (mEq/L)	Whole group	4.5 [4.1 - 4.9]	4.3 [4.0 - 4.6]	4.2 [3.9 - 4.4]	0.164
	≥ |8|mEq/L	4.4 [3.7 - 5.2]	4.4 [4.2 - 4.6]	4.1 [3.9 - 4.3]	0.819
	< |8|mEq/L	4.5 [4.1 - 4.9]	4.3 [4.0 - 4.7]	4.2 [3.9 - 4.5]	0.227
Na^+^ - Cl^-^ (mEq/L)	Whole group	36.0 [32.0 - 40.0]	36.5 [34.0 - 41.0]	37.5 [32.8 - 41.2]	0.104
	≥ |8|mEq/L	33.3 [2.8 - 34.8]	38.5 [36.5 - 40.5]	34.0 [32.0 - 37.0]	0.074
	< |8|mEq/L	36.0 [33.3 - 40.0]	35.3 [34.0 - 41.0]	38.0 [34.5 - 41.5]	0.071
Temperature (ºCelsius)	Whole group	36.2 [36.7 - 36.6]	35.7 [35.2 - 36.4]	36.0 [35.6 - 36.6]	0.495
	≥ |8|mEq/L	36.7 [35.6 - 36.3]	35.6 [35.1 - 36.0]	36.2 [35.6 - 36.7]	0.147
	< |8|mEq/L	36.2 [35.6 - 36.6]	36.2 [35.5 - 36.6]	36.0 [35.6 - 36.6]	0.888
Diuresis (mL)†	Whole group	0 [0 - 0]	40 [0 - 465]	191 [0 - 875]	< 0.001
	≥ |8|mEq/L	0 [0 - 0]	25 [0 - 463]	0‡ [0 - 600]	0.180
	< |8|mEq/L	0 [0 - 0]	55 [0 - 420]	280 [10 - 895]	0.006
Fluid balance (mL)†	Whole group	0 [0 - 0]	0 [0 - 0]	0 [-720 - 969]	0.872
	≥ |8|mEq/L	0 [0 - 0]	0 [0 -105]	0‡ [-310 - 796]	0.446
	< |8|mEq/L	0 [0 - 0]	0 [0 -0]	0 [-905 - 994]	0.861

Na^+^ - sodium; CRRT - continuous renal replacement therapy; PaCO_2_- partial pressure of carbon dioxide; SBE - standard base excess; HCO_3_ - bicarbonate; K^+^ - potassium; Na^+^ - Cl^-^ - sodiumchloride.

* p value of the Friedman’s test, comparing the variables trough the time.

† Diuresis and fluid balance are cumulative from the continuous renal replacement therapy beginning.

‡ p > 0.05 versus < |8|mEq/L group (Wilcoxon’s test). The results are expressed as the median (25% - 75%).

**Table 4:** Continuous renal replacement therapy data during the first 24 hours

	**[Na^+^] 24 hours variation**	**CRRT initiation**	**12 hours**	**24 hours**	p value*
Blood flow (mL/minute)	Whole group	180 [150 - 180]	180 [150 - 180]	180 [150 - 180]	0.651
	≥ |8| mEq/L	180 [158 - 180]	180 [180 - 180]	180 [150 - 180]	0.589
	< |8| mEq/L	180 [158 - 180]	180 [150 - 195]	180 [150 - 180]	0.958
Dialysate flow (mL/hour)	Whole group	1000 [1000 - 1200]	1000 [1000 - 1200]	1000 [1000 - 1200]	-----
	≥ |8| mEq/L	0 [0 - 0]	0 [0 - 0]	0 [0 - 0]	-----
	< |8| mEq/L	1000 [1000 - 1300]	1000 [1000 - 1300]	1000 [1000 - 1300]	-----
Replacement fluid flow (mL/hour)	Whole group	2000 [1500 - 2500]	2000 [1500 - 2500]	2500 [1500 - 2500]	-----
	≥ |8| mEq/L	2000 [1850 - 2000]	2000 [1850 - 2000]	2000 [1850 - 2000]	-----
	< |8| mEq/L	2000 [1500 - 2500]	2000 [1500 - 2500]	2000 [1500 - 2500]	-----
ACD-A 2.2% flow (mL/hour)	Whole group	180 [180 - 180]	180 [180 - 180]	180 [180 - 182]	0.223
	≥ |8| mEq/L	180 [180 - 180]	180 [180 - 180]	180 [180 - 180]	1.000
	< |8| mEq/L	180 [180 - 180]	180 [180 - 180]	180 [180 - 190]	0.368
Citrate 4% flow (mL/hour)	Whole group	150 [140 - 160]	150 [140 - 160]	150 [140 - 160]	0.692
	≥ |8| mEq/L	160 [145 - 170]	140 [135 - 145]	150 [140 - 160]	0.264
	< |8| mEq/L	150 [140 - 160]	160 [140 - 160]	150 [140 - 160]	0.973
Volume of lavage (mL/hour)	Whole group	300 [225 - 300]	300 [300 - 300]	300 [188 - 300]	0.819
	≥ |8| mEq/L	450 [375 - 525]	200 [150 - 250]	300 [200 - 300]	0.368
	< |8| mEq/L	300 [150 - 300]	300 [300 - 300]	300 [225 - 300]	0.692
Affluent fluid [Na^+^] (mEq/L)	Whole group	138 [127 - 140]	138 [127 - 140]	139 [127 - 140]	1.000
	≥ |8| mEq/L	140 [125 - 142]	140 [125 - 142]	140 † [125 - 142]	1.000
	< |8| mEq/L	137 [128 - 140]	137 [128 - 140]	137 [128 - 140]	1.000
Ultrafiltration rate (mL/hour)	Whole group	100 [100 - 164]	150 [100 -2 00]	110 [100 - 164]	0.257
	≥ |8| mEq/L	75 [50 - 125]	150 [130 - 164]	100 [100 - 100]	0.368
	< |8| mEq/L	100 [100 - 164]	150 [100 - 200]	150 [100 - 200]	0.612
Effluent flow‡ (mL/hour)	Whole group	2340 [1975 - 2794]	2270 [1808 - 2745]	2290 [1800 - 2730]	0.918
	≥ |8|mEq/L	2225 [2010 - 3123]	2265 [1824 - 2655]	2360 [1910 - 2600]	0.895
	< |8|mEq/L	2385 [1925 - 2784]	2270 [1835 - 2715]	2290 [1800 - 2735]	0.964

Na^+^ - sodium; CRRT - continuous renal replacement therapy; ACD-A - acid citrate dextrose - formula A.

* p value of the Friedman’s test, comparing the variables trough the time.

† > 0.05 versus < |8|mEq/L group (Wilcoxon’s test).

‡ Effluent flow equalizes the sum of dialysate, replacement fluid, 2.2% ACD-A, 4% citrate, lavage and ultrafiltration rate. The results are expressed as the median (25% - 75%).

**Figure 3:**
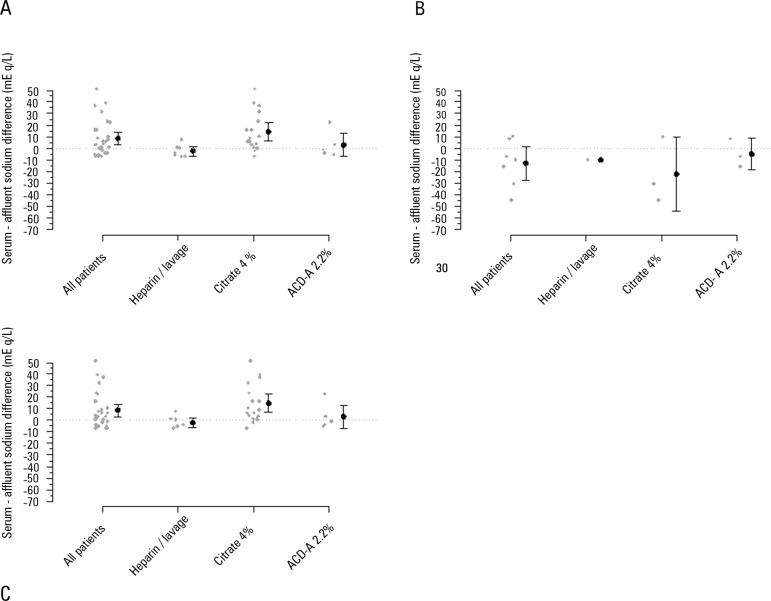
Difference between serum and affluent sodium after 24 hours of continuous renal replacement therapy. A) All sessions of continuous venous-venous hemofiltration. B) Sessions where the sodium variation was ≥ |8|mEq/L. C) Session where the sodium variation was < |8|mEq/L. ACD-A - acid citrate dextrose - formula A. Gray points represent the session's individual variations. Black points and bars represent the mean variation and 95% confidence interval.

**Figure 4:**
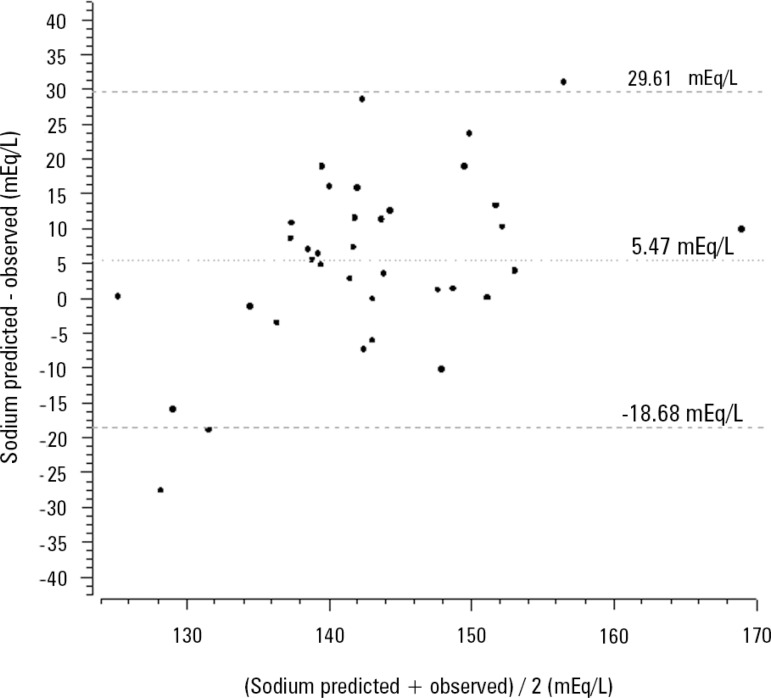
Bland-Altman diagram showing the agreement between the serum sodium predicted using the total admixture formula and serum sodium after 24 hours of continuous renal replacement therapy initiation.

At CRRT initiation, the prescribed affluent sodium was 2 [1 - 6], 10[-25 - 1], and 4 [-12 - 8] mEq/L lower than the serum sodium in sessions that used heparin or lavage, 4% citrate and 2.2% ACD-A, respectively, to enhance filter protection. Figure 3A to C shows the difference between serum sodium at the end of 24 hours of the CRRT session and the affluent prescribed sodium concentration. Taking into account only CRRT sessions (28 sessions) that used citrate (2.2% ACD-A or 4% citrate), a 24-hour serum sodium variation of 1.5 [-0.5 - 5.5] mEq/L with an initial serum sodium concentration of 140 [136 - 144] mEq/L and sodium in the solution of 133 [125 - 139] mEq/L was observed. Among these CRRT sessions, when only initially hypernatremic patients were observed (total of six sessions) (serum sodium > 145mEq/L), the sodium variation, serum concentration, and solution concentration were 9.5 [4.8 - 14.0] mEq/L, 167 [159 - 175] mEq/L and 165 [160 - 169] mEq/L, respectively. Figure 4 shows the poor agreement between the observed serum sodium at the end of 24 hours of CRRT and the expected serum sodium calculated using the perfect admixture formula.

## DISCUSSION

This study showed that in the CRRT sessions, serum sodium variations ≥ |8|mEq/L occurred more frequently in severely ill patients with hypernatremia at the time of CRRT initiation. The perfect admixture formula for sodium prediction is not accurate, and the use of 4% citrate or 2.2% ACD-A as anticoagulants was not associated with higher serum sodium variations.

A large variation in serum sodium concentration during extracorporeal therapy is potentially dangerous to critically ill patients, mainly because a large osmolality variation is associated with hemodynamic instability^(17)^ and encephalic water interstitial changes.^(18)^ Thus, the main finding of this study supports that patients with hyperfnatremia at CRRT initiation are more prone to present dangerous serum sodium concentration variations within the first 24 hours of CRRT and should be monitored closely to avoid iatrogenic insults. Despite the recognition of pre-CRRT hypernatremia as a mortality predictor,^(19)^ it is not consistently associated with serum sodium variations after CRRT initiation.^(20)^ Furthermore, hypernatremic patients are more severely ill and present more organ dysfunction than non-hypernatremic patients, as demonstrated by other studies.^(21)^

The use of citrate-related anticoagulants significantly enhances filter lifespan compared with unfractionated heparin.^(22)^ However, the sodium load measured in citrate salts is very high, which can worsen the serum sodium prediction after CRRT initiation. The practice of prescribing affluent sodium lower than the serum sodium concentration when using citrate-related anticoagulants is frequent.^(9,23)^ In the present study, the sodium concentration in affluent solutions was 10mEq/L lower than the serum sodium concentration when using 4% citrate and 4mEq/L lower than he serum sodium concentration when using 2.2% ACD-A, a finding that enables the use of citrate-related anticoagulants without a significant effect in sodium variation over 24 hour observation period. However, the interpretation of these data regarding citrate and sodium variations must be taken into account as this study is underpowered to detect differences among the anticoagulant groups. In hypernatremic patients (serum sodium > 145mEq/L) who used 4% citrate or 2.2% ACD-A, the prescribed sodium in the affluent fluid was similar to the serum sodium of the patients (see the results section) to minimize the serum concentration reduction; however, the serum sodium concentration decrease during the first 24 hours of CRRT was approximately 9mEq/L, a finding that elicits concern when prescribing CRRT using any citrate formulation in hypernatremic patients.

The mathematical prediction of the 24-hour serum sodium behavior using the perfect admixture formula presented in this manuscript is not suitable for bedside use because the agreement with observed serum sodium was poor. However, there are some hypotheses in response to these results. First, the equilibrium of electrolytes across membranes, as low weight molecules, is theoretically perfect; however, mass transfer can be affected by many other erratic factors; for instance, different charges in high weight organic molecules, which is also known as the Gibbs-Donnan effect.^(24)^ Thus, sodium variation may be unpredictable during medium to long time periods of CRRT.

Another confounder when trying to predict sodium during extracorporeal therapies is the non-homogeneous mixing inside the circuit. It is hypothesized that approximately one meter inside the circuit, a given solution added to the blood can run in a different phase from the blood, passing through the filter as an independent solution, a finding that can lead to unpredictable filtration.^(25)^ Third, in this model, the sieving coefficient of citrate was considered to be 1 for model simplification; however, this finding may not hold true because the sieving coefficient is actually approximately 0.9.

This study has some limitations. First, the small sample size could potentially reduce the sensitivity of the analyses for relevant associations. Second, there were no patients with severe hyponatremia, and thus, this subgroup was not well evaluated. Third, the outcome measure (sodium variation ≥ 8mEq/L) might be too strict, and smaller variations could have occurred using citrate-based anticoagulation; however, we believe they are not very relevant to clinical practice. Fourth, this study represents the practice of only two centers, which might jeopardize external validity. Fifth, one may be concerned that there were no stepwise adjustments of the sodium concentration of dialysate/replacement fluids during CRRT in hypernatremic patients in this population because according to the study inclusion criteria, patients were retrieved if the affluent fluid prescription was not modified for 24 hours; however the authors chose to do this so that the internal validity related to statistical analysis was not compromised. Sixth, the disease severity of patients was not very high and the dynamics among different body compartments in more severely ill patients could potentially modify the results presented in this study.

## CONCLUSIONS

Hypernatremia at the time of continuous renal replacement therapy initiation is an important factor that is associated with clinically significant serum sodium variations, and the intensivist should be aware of this finding when continuous renal replacement therapy is started. The use of 4% citrate or 2.2% acid citrate dextrose - formula A as anticoagulants is not associated with serum sodium variations ≥ 8mEq/L if the initial affluent fluid prescription considers its sodium content in advance. The use of a specific mathematical calculation could not predict the 24-hour sodium variation.

### Authors' contributions

All of the authors significantly contributed to this manuscript, including the study conception (M Park, TG Romano), data acquisition (TG Romano, M Park, CPB Martins), data analysis and interpretation (M Park, TG Romano, BAMP Besen), drafting of the manuscript (M Park, TG Romano, BAMP Besen, FG Zampieri, PV Mendes), revision of the manuscript for important intellectual content (all authors), and approval of the final copy (all authors).
